# Early Diagnosis of Heterotopic Pregnancy in a Primigravid Without Risk Factors in the Emergency Department

**DOI:** 10.5811/cpcem.2019.1.41312

**Published:** 2019-02-26

**Authors:** Rachel O’Donnell, Elizabeth Siacunco, Daniel Quesada, Kieron Barkataki, Phillip Aguìñiga-Navarrete

**Affiliations:** * Kern Medical, Department of Emergency Medicine, Bakersfield, California; † LAC+USC Medical Center, Los Angeles, California; ‡ UCLA Health Sciences, Department of Emergency Medicine, Bakersfield, California

## CASE PRESENTATION

**Image 1:**
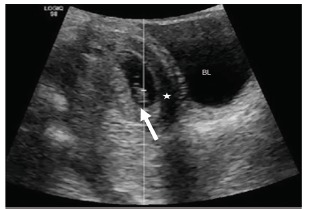
Viable intrauterine pregnancy (arrow) with free fluid in the pelvis (star). *BL*, bladder.

**Image 2:**
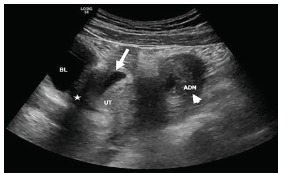
Transabdominal ultrasonography revealing a gestational sac in the uterus (arrow) and a complex mass with central hypoechogenicity in the left adnexa (arrowhead), suggestive of an extrauterine pregnancy. *UT*, uterus; *ADN*, adnexa; *BL*, bladder. Star = free fluid.

A 19-year-old primigravida female presented with three weeks of intermittent suprapubic and left lower quadrant (LLQ) abdominal pain, worsening in the prior 24 hours, associated with nausea and vomiting at the time of presentation. Her last normal menstrual period was approximately 17 weeks prior to presentation, but she reported some vaginal spotting nine weeks ago. Abdominal exam revealed diffuse tenderness to palpation, worse in the LLQ, without peritoneal signs. A point-of-care ultrasound (POCUS) showed an intrauterine pregnancy (IUP). However, the patient’s persistent unilateral pain was concerning. Therefore, a formal pelvic ultrasound was performed, which revealed an IUP at seven weeks gestation, including an anechoic region with free fluid in the pelvis (Image 1), and a left adnexal complex mass suspicious for extrauterine pregnancy (Image 2). She subsequently underwent a laparoscopic left salpingectomy for a ruptured ectopic pregnancy. The IUP was unaffected.

## DISCUSSION

While heterotopic pregnancy (HP) is rare, its frequency has increased with the advent of fertility treatment and reproductive technologies.^1^ Other risk factors include history of ectopic pregnancy, pelvic inflammatory disease, and prior tubal surgery.^2^ Our patient had no risk factors. While ectopic pregnancy is a leading case of maternal death, HP has a good prognosis if diagnosed early.^3^ After treatment of an extrauterine pregnancy, more than half proceed with an otherwise-uneventful IUP to term.^2^ However, its diagnosis is challenging, and many of these patients initially present to the emergency department with abdominal pain, vaginal bleeding, or both. With the emergence of POCUS, emergency physicians are trained to identify IUPs.^4^ A study concluded that emergency physicians were able to successfully use ultrasound to rule out ectopic pregnancy by locating an established IUP with embryonic structures.^5^ Thus, the presence of an IUP can mask a concomitant extrauterine pregnancy, delaying its diagnosis and potentially resulting in life-threatening hemorrhage. This case illustrates the need for emergency physicians to maintain a high index of suspicion for HP even in patients without risk factors, and the need to evaluate the entire pelvis despite an IUP.

**CPC-EM Capsule**

What do we already know about this clinical entity?

*Heterotopic pregnancy is rare, and most cases are seen in patients undergoing infertility treatment. Additional risk factors are similar to those of an ectopic pregnancy*.

What is the major impact of the image(s)?

*Heterotopic pregnancy can be seen in those without obvious risk factors. A viable intrauterine pregnancy (IUP) does not completely rule out a concurrent ectopic pregnancy*.

How might this improve emergency medicine practice?

*Most EM physicians rule out an ectopic pregnancy if an IUP is seen on bedside ultrasound. This is a reminder to maintain a high index of suspicion for a heterotopic pregnancy despite a viable IUP*.

## References

[1] Tal J, Haddad S, Gordon N (1996). Heterotopic pregnancy after ovulation induction and assisted reproductive technologies: a literature review from 1971 to 1993. Fertil Steril.

[2] Barrenetxea G, Barinaga-Rementeria L, Lopez de Larruzea A (2007). Heterotopic pregnancy: two cases and a comparative review. Fertil Steril.

[3] Schneider J, Berger CJ, Cattell C (1977). Maternal mortality due to ectopic pregnancy. A review of 102 deaths. Obstet Gyneol.

[4] Michalke J (2012). An overview of emergency ultrasound in the United States. World J Emerg Med.

[5] Durham B, Lane B, Burbridge L (1997). Pelvic ultrasound performed by emergency physicians for the detection of ectopic pregnancy in complicated first-trimester pregnancies. Ann Emerg Med.

